# Optic disc topography in normal Indian eyes using spectral domain optical coherence tomography

**DOI:** 10.4103/0301-4738.73716

**Published:** 2011

**Authors:** Tarannum Mansoori, Kalluri Viswanath, Nagalla Balakrishna

**Affiliations:** Department of Glaucoma, Pushpagiri Eye Institute, 241, Uma Plaza, West Marredpally, Secunderabad, Andhra Pradesh, India; 1 Department of Biostatistics, National Institute of Nutrition, Hyderabad, Andhra Pradesh, India

**Keywords:** Optic nerve head topography, spectral domain optical coherence topography, Indian eyes

## Abstract

**Purpose:**

The aim was to study optic nerve head (ONH) parameters in normal Indian eyes using spectral domain optical coherence tomography (OCT)/scanning laser ophthamoscope (SLO).

**Materials and Methods:**

One hundred and fifty-seven eyes of 157 normal subjects of various age groups underwent ONH imaging with spectral OCT/SLO and the parameters obtained were correlated with disc size. The effect of age, gender, and refractive error on various ONH parameters were also studied.

**Results:**

The mean optic disc area was 3.36 ± 0.64 mm^2^ (range, 2.13–5.08 mm^2^), mean rim area was 2.49 ± 0.58 mm^2^ (range, 1.20–3.62 mm^2^), and mean cup area was 1.10 ± 0.75 mm^2^ (range, 0–3.07 mm^2^). The disc area showed significant positive correlation with the rim area, cup area, horizontal cup disc ratio, vertical cup disc ratio, cup disc area ratio, mean cup depth, and maximum cup depth (*P* < 0.001). Neither gender nor refractive error showed any significant difference in various ONH parameters. ONH parameters did not show significant change with age except for rim area which declined with the advancing age (*r* = –0.25, *P* < 0.001).

**Conclusions:**

The quantitative measurement of ONH topography obtained with this study provides a normative database for an Indian population with spectral OCT/SLO. As optic disc area influences ONH topography, disc size should to be considered when evaluating optic disc for progressive optic neuropathies such as glaucoma.

Optic nerve head (ONH) evaluation is essential for the diagnosis and management of glaucoma and other optic nerve anomalies. It can be assessed by clinical examination or stereo photographs which are subjective and qualitative techniques with high interobserver variability. Hence, accurate and objective method for detecting early disc abnormality and their progression may help in early diagnosis and monitoring of glaucoma. Several imaging modalities are employed in clinical practice to obtain objective and quantitative estimation of ONH topography.[1–9] Large variation in disc size within a population[2] and among populations[6] has been reported. Also, the techniques used for ONH measurement can provide different estimates of disc size limiting comparison among studies.[1–9]

Optical coherence tomography (OCT) is a noncontact, noninvasive transpupillary imaging technique which provides *in vivo*, high-resolution, cross-sectional images to visualize and measure anatomic layers of retina and ONH. It measures echo time delay and intensity of backscattered light from various retinal layers using an optical correlation technique known as Michelson low coherence interferometry. In the new generation spectral OCT/scanning laser ophthamoscope (SLO) (OPKO/OTI, V2.26, Miami, Florida USA), reflectance interference between the reference arm and retina at each A-scan location is Fourier transformed to simultaneously acquire all points along the depth of A-scan without reference arm movement. As each A-scan is acquired all at once, the acquisition rate is much higher at 27,000 A-scans/second as opposed to the time domain OCT (Stratus OCT) rate of 400 A-scans/second. The fast acquisition rate allows for much faster scanning time, reducing motion artifacts and enabling denser patterns across the ONH with improved axial resolution of <6 µm when compared to Stratus OCT (8–10 µm).[10]

Topography values in normal Indian population are available with Stratus OCT.[3,5] To the best of our knowledge, a normative database for ONH parameters for spectral OCT/SLO for Indian eyes is unavailable in literature. This study was undertaken to assess ONH topography measurements in an Indian population with normal eyes using spectral OCT/SLO. In addition, the effect of age, gender, refractive error, and disc size on various ONH parameters was also studied.

## Materials and Methods

This prospective study included 210 volunteers from the Institute staff and patients who had come to the Institute for refractive error correction between March 2009 and October 2009. The study followed the guidelines contained in the Declaration of Helsinki. After informed consent, one randomly selected eye per subject was included in the study. All subjects were Asian Indians.

Each participant underwent complete ophthalmic examination, including best corrected visual acuity (BCVA), refraction, slit lamp biomicroscopy, intraocular pressure (IOP) measurement by the Goldmann applanation tonometer, gonioscopy by the Volk four-mirror Sussman goniolens, optic disc and stereoscopic fundus evaluation with a +78 D lens and achromatic automated perimetry using the Swedish interactive threshold algorithm (SITA) Standard 24-2 program with a Humphrey visual field analyzer (Carl Zeiss Meditec, Dublin, California, USA).

For inclusion, all subjects had normal ocular examination, IOP < 21 mmHg, no past history of raised IOP, BCVA ≥ 20/30, refractive error within ± 3 diopters (D) sphere and ± 1.5 D cylinder, clear ocular media (nuclear opalescence, nuclear color, and cortical changes up to grade 2 on lens opacity classification system III),[11] open angles on gonioscopy, normal optic disc appearance, i.e., absence of glaucomatous optic neuropathy (defined as no vertical cup –disc asymmetry of > 0.2 between 2 eyes, intact neuroretinal rim [NRR] without peripapillary hemorrhages, notches, localized pallor, or retinal nerve fiber layer defect), and normal and reliable (fixation losses ≤ 20%, false positives, and false negatives ≤ 33%) visual field. Normal visual field indices were defined as mean deviation and pattern standard deviation within 95% confidence limits and glaucoma hemifield test “within normal limits.” Subjects with a family history of glaucoma, intraocular surgery, ocular trauma, neurological disease, uveitis, corneal, retinal, or macular pathology, and abnormal disc-like tilted discs with parapapillary atrophy were excluded. After dilation with the 1% tropicamide eye-drop, ONH images were acquired by a single operator (TM) with spectral OCT/SLO using the optic nerve topography scan mode. The subjects were asked to look at the internal fixation target and the location of ONH was observed on the SLO image to ensure proper positioning of the scan. The topography stack covers an area of 5.5 × 5.5 mm with a depth of 2 mm. A three-dimensional tomographic image of ONH was generated from a stack of sequential OCT and confocal SLO images. The operator made sure that the center of the optic disc was in the center of the SLO image. The device automatically determines the disc margin as the end of the retinal pigment epithelium (RPE) and a straight red line connects the edge of RPE. A parallel yellow line is constructed at a fixed offset of 150 µm anterior to the plane of RPE reflection. Measurements below this line are defined as the disc cup and measurements above were considered the NRR. It is important to note that the cup area in spectral OCT/SLO is measured independent of disc area. Demarcation of the edge of RPE can be manually adjusted to improve the outline of the disc margin. However, we did not use the manual algorithm to avoid introducing a subjective component to our analysis. The software analyses the data and creates a report on disc area, cup area, rim area, cup/disc horizontal ratio (CDHR – ratio of the longest horizontal line across the cup to the longest horizontal line across the disc), cup/disc vertical ratio (CDVR – ratio of the longest vertical line across the cup to the longest vertical line across the disc), cup/disc area ratio (CDAR – ratio of cup area to disc area), mean cup depth, and maximum cup depth. Only images with a focused SLO image of ONH, with signal strength ≥ 7, were considered to be of acceptable quality. Scans where appropriate automated detection of the ONH margin failed and hence required manual correction for recognition of disc margin were not included in the study. To determine the intrasession reproducibility of ONH parameters, 20 study volunteers from staff of the Institute underwent ONH imaging three times in a day with a gap of 30 min between each scan. Statistical analysis was performed using SPSS software, version 15 (SPSS Inc., Chicago, IL, USA). Descriptive analysis including mean and standard deviation (SD), 1^st^, 5^th^, 50^th^, and 95^th^ percentiles were calculated for all ONH parameters. Correlation between various ONH variables and association between refractive error and ONH parameters were evaluated by Pearson’s correlation coefficient. Student’s *t* – test was used to compare mean differences of ONH parameters between gender. Linear regression analysis was used to determine the effect of age on various ONH parameters and disc area on ONH variables. Previous reports[2] estimate the mean morphometric optic disc size as 2.58 ± 0.65 mm^2^ in the Indian population. The sample size was determined with power of 80% using the formula $n\;=\;2{\left(Z_{a}\;+\;Z_{b}\right)}^{2}S^{2}/d^{2}.$.[3] Considering the SD of 0.65, the minimum sample size calculated was 40. Intraclass correlation coefficient (ICC) and coefficient of variation (CV) were calculated to compare the three measurements obtained on the same day for each ONH parameter. Statistical significance was shown at *P* < 0.05.

## Results

A total of 157 scans, adequately recognized and measured by the automated disc analysis algorithm, were qualified for inclusion in the study. Of the 210 scans, 53 scans did not qualify for inclusion due to following reasons: 40 (76%) scans had inappropriate automated recognition of disc margin and required manual correction, 11 (21%) scans had imaging artifacts interfering with disc margin recognition, and in 2 (4%) scans the software could not recognize the automated ONH analysis algorithm using a cup offset of 150 µm in the small disc with no cup.

The mean ± SD age of the subjects was 41.3 ± 15.5 years (range, 13–79). There were 55 (35%) males, mean age 41.5 ± 16.9 years, and 102 (65%) females, mean age 39.6 ± 14.1 years. Eighty-eight (56.05%) eyes were emmetropic, 39 (24.84%) myopic, and 30 (19.11%) hypermetropic. The mean spherical refractive error was 0.97 ± 0.8 D and the mean astigmatic error was 0.6 ± 0.2 D. The mean ± SD of disc area was 3.36 ± 0.6 mm^2^ [Table 1]. There was no gender-related difference in the disc area [Table 2]. The disc area did not show significant correlation with the refractive error (*P* = 0.24) and age (*r* = –0.12, *P* = 0.12). There was significant positive correlation of disc area with cup area, rim area, CDHR, CDVR, CDAR, mean cup depth, and maximum cup depth [Table 3].

**Table 1:** Mean ± standard deviation (SD), range, 95% confidence interval for mean, first, fifth, fiftieth and ninety fifth percentiles of various optic nerve head (ONH) parameters

ONH parameters	Mean ± SD	Range	95% confidence interval for mean	1^st^ percentile	5^th^ percentile	50^th^ percentile	95^th^ percentile
Disc area (mm^2^)	3.36 ± 0.64	2.13 – 5.08	3.27 – 3.69	2.14	2.42	3.37	4.85
Cup area (mm^2^)	1.1 ± 0.75	0.0 – 3.07	0.98 – 1.22	0.0	0.0	1.63	3.82
Rim area (mm^2^)	2.49 ± 0.58	1.20 – 3.62	2.4 – 2.58	0.58	0.82	1.65	2.74
Cup/disc horizontal ratio	0.5 ± 0.23	0.0 – 0.88	0.46 – 0.53	0.0	0.0	0.81	0.9
Cup/disc vertical ratio	0.5 ± 0.21	0.0 – 0.81	0.47 – 0.54	0.0	0.0	0.71	0.88
Cup/disc area ratio	0.29 ± 0.18	0.0 – 0.68	0.26 – 0.32	0.0	0.0	0.51	0.81
Mean cup depth (mm)	0.18 ± 0.11	0.0 – 0.53	0.16 – 0.19	0.0	0.0	0.14	0.3
Maximum cup depth (mm)	0.36 ± 0.21	0.0 – 0.98	0.33 – 0.4	0.0	0.0	0.37	0.64

**Table 2:** Gender based comparison (Mean ± standard deviation) of Optic nerve head topography parameters in study population

Optic disc parameters	Male	Female	*P* value
Disc area (mm^2^)	3.31 ± 0.7	3.29 ± 0.6	0.5
Cup area (mm^2^)	1.2 ± 0.7	1.2 ± 0.7	0.1
Rim area (mm^2^)	2.5 ± 0.5	2.5 ± 0.6	0.1
Cup/disc horizontal ratio	0.53 ± 0.2	0.5 ± 0.2	0.3
Cup/Disc vertical ratio	0.5 ± 0.2	0.47 ± 0.2	0.1
Cup/Disc area ratio	0.32 ± 0.2	0.27 ± 0.2	0.1
Mean cup depth (mm)	0.19 ± 0.1	0.17 ± 0.1	0.3
Maximum cup depth (mm)	0.39 ± 0.2	0.34 ± 0.2	0.15

**Table 3:** Effect of optic disc area (Y) on optic disc variables (X)

Variables	Equation	*P* value	Correlation coefficient (R)
Cup area (mm^2^)	Y= 2.97 + 0.57 X	<0.001	0.63
Rim area (mm^2^)	Y= 2.94 + 0.26 X	<0.001	0.25
Cup / disc horizontal ratio	Y= 2.89 + 1.41 X	<0.001	0.45
Cup / disc vertical ratio	Y= 2.71 + 1.72 X	<0.001	0.48
Cup / disc area ratio	Y= 3.09 + 1.70 X	<0.001	0.45
Mean cup depth (mm)	Y= 3.21 + 2.15 X	<0.001	0.38
Maximum cup depth (mm)	Y= 3.20 + 1.09 X	<0.001	0.37

The mean ± SD of the cup area was 1.1 ± 0.7 mm^2^ [Table 1]. There was no gender-related difference in cup area [Table 2]. The cup area did not show significant correlation with refractive error (*P* = 0.54) and age (*r* = 0.09, *P* = 0.3). It showed significant positive correlation with the disc area [Table 3].

The mean ± SD of the NRR area was 2.48 ± 0.6 mm^2^ [Table 1]. There was no gender-related difference in the NRR area [Table 2] and it did not show significant correlation with the refractive error (*P* = 0.34). There was a decrease in the NRR area with the increasing age (*r* = –0.25, *P* < 0.001). The NRR area showed negative correlation with the cup area (rim area = 2.97 – 0.43 cup area, *r* = –0.54, *P* < 0.001) and significant positive correlation with the disc area [Table 3]. There was no gender-related difference in CDR [Table 2]. No significant association between refractive error and CDR parameters measured was detected (*P* = 0.5). There was no effect of age on CHDR (*r* = 0.83, *P* = 0.3), CHVR (*r* = 0.11, *P* = 0.2), and CDAR (*r* = 0.13, *P* = 0.1). There was no effect of gender [Table 2], refractive error (*P* = 0.6) or age on the mean cup depth (*r* = 0.04, *P* = 0.6) and maximum cup depth (*r* = 0.03, *P* = 1.0).

Intrasession ICC for ONH parameters ranged from 0.999 for the disc area to 0.974 for the rim area. CV ranged from 11.8% for the disc area to 30.4% for CDAR.

## Discussion

The evaluation of the ONH morphology by various methods in an Indian population with normal eyes provides different estimates of optic disc size.[1–5] With the development of new imaging technologies such as spectral OCT/SLO, it is necessary to have ethnic specific normative database for ONH topography. In a study on the reproducibility of ONH measurements with spectral domain OCT (RTVue) in 60 healthy and 76 glaucomatous eyes, CV ranged from 0.27% and 0.4%, respectively, for disc area to 29.1% and 21.3%, respectively, for cup volume.[12] As the reproducibility of ONH parameters with spectral OCT/SLO has not been reported before, we calculated the intrasession reproducibility in 20 volunteers and found excellent ICC for all ONH parameters. As it was not the purpose of the study to measure the reproducibility of OCT and the sample size was small, further studies with larger sample size may establish intrasession and intersession reproducibility of ONH parameter measurements with spectral OCT/SLO. The Rotterdam Study on white people demonstrated that the mean optic disc area was on an average 3.2% larger in males than in females.[13] Varma *et al*.[6] found that male subjects had optic discs that were 2–3 % larger than those of female subjects. However, this difference in the size of the disc was small, and probably has questionable clinical importance. In the present study, we found no significant difference between gender with regard to various ONH parameters as reported by other authors in healthy Indian eyes.[1–3] Jonas *et al*.[14] reported that the optic disc size depends on the refractive error, with an increase in the disc size in highly myopic eyes beyond –8 D and a decrease in highly hyperopic eyes beyond +4 D. In our study, refractive error ranged between ±3 D of sphere and for this range of refraction we found no statistically significant difference in various ONH parameters. Similar findings were reported by Dacosta *et al*.[3] (range, -5 to +3 D) and Jonas *et al*.[2] (range, –4.5 to +2.5 D) in normal Indian eyes. Further studies are needed to determine whether ONH parameters differ in high myopic and high hyperopic eyes.

Variations in the optic nerve morphology among different races have been reported previously using optic disc photographs, planimetry and imaging techniques[1–9] [Table 4]. The optic disc area in our study was comparable to that found by Sekhar *et al*.[1] in normal Indian eyes using planimetric technique (*P* = 0.089) and showed higher values when compared to other studies.[2–8] This discrepancy could be due to the difference in sample size, ethnic variation, different techniques, and analysis protocol used. The disc area measured in this study was larger than that reported by another studies in normal Indian eyes using Stratus OCT,[3,5] which may be attributed to the inclusion of more number of eyes with large disc area in the current study [Table 1]. In our study, the disc area showed an inter- individual variation of 1: 2.38. A study in white eyes showed an inter- individual variation in disc area of 0.8 to 5.54 mm^2^.[15] Histological[16] and OCT[17] studies have shown that rim area decreases with aging while another study did not show any change in ONH topography[13] In our study, rim area showed decline with age which was statistically significant (*r* = - 0.25, *P* < 0.001). However the correlation ‘r’ was very low and hence the correlation is weak and not clinically significant. Also, the cup area did not show increase with aging, suggesting stability. The best way to know the variation in ONH with age is to follow up same normal subjects longitudinally. In this study, rim area showed inter individual variation of 1:3.02. As rim area is derived from disc area and cup area, an increase in rim area was seen with increasing disc size, as reported in previous studies.[15,18,19]

**Table 4:** Disc area in normal eyes as reported in various studies

Authors	Population studied	Method of disc examination	Number of subjects	Mean disc area in mm^2^ (standard deviation)
Present study	Indian	Spectral domain OCT	157	3.36 (0.64)
Sekhar *et al*.[1]	Indian	Planimetry	143	3.37 (0.68)
Jonas *et al*.[2]	Indian	Stereophotographs	70	2.58 (0.65)
Dacosta *et al*.[3]	Indian	Stratus OCT	150	2.63 (0.55)
Agarwal *et al*.[4]	Indian	HRT II	275	2.34(0.47)
Varma *et al*.[6]	Blacks	Stereophotographs with image analyzer	2903	2.94(0.74)
	Whites		3475	2.63(0.46)
Funaki *et al*.[7]	Japanese	Scanning laser polarimetry	60	2.2(0.55)
R Ramakrishnan *et al*.[5]	Indian	OCT 3	82	2.37 (0.51)
		Planimetry	82	2.83 (0.62)
Medeiros *et al*.[8]	Caucasian	Stratus OCT	78	2.35 (0.51)
Marsh *et al*.[9]	Whites	Stratus OCT	212	2.17 (95% CI 1.56-2.99)
	Hispanic			2.33 (95% CI 1.67-3.09)
	African - American			2.49 (95% CI 1.8-3.24)

The cup area showed inter- individual variation in the eyes examined (range, 0-3.07mm^2^). It showed significant positive correlation with disc area and negative correlation with rim area. Clinical importance of this finding is that in early glaucoma, optic nerve damage may be overlooked in small optic disc with relatively small cup, if one does not take into account that small disc normally have small or no optic cup and a large optic cup can be physiological in eyes with large ONH which can be mistaken for glaucoma.

The CDR showed significant positive correlation with disc area. Although it is easy to perform CDR measurements clinically, the measurement has limited value if not adjusted for disc size. The drawback of spectral domain OCT is that it does not adjust scan size to compensate for refractive error, corneal curvature or axial length.[20] Limitation of this study is that the participants were selected from those subjects who had come to the Institute and subjects with tilted disc and parapapillary atrophy were not included in the study. Hence, the data cannot be extrapolated to the general population.

To summarize, this study reports normative database for ONH parameters in healthy Indian eyes using spectral OCT /SLO. Further studies in different population groups using same methodology may answer the question whether disc area shows ethnic variation.
